# Antimicrobial peptide Pt5-1c exerts potent immunomodulatory and antiviral effects by facilitating cellular delivery of cGAMP

**DOI:** 10.3389/fimmu.2026.1909072

**Published:** 2026-09-11

**Authors:** Xuelian Zhao, Zetao Ding, Jingwei Zhang, Shuran Yang, Yang Zhu, Yuan Tang, Hao Wang, Beibei Chen, Chanyuan Guo, Rijun Zhang, Dayong Si, Xubiao Wei

**Affiliations:** State Key Laboratory of Animal Nutrition and Feeding, College of Animal Science and Technology, China Agricultural University, Beijing, China

**Keywords:** antimicrobial peptide, antiviral effects, cGAMP, cGAS-STING signaling, immunology

## Abstract

The cGAS-STING signaling axis constitutes a pivotal defense mechanism in innate immunity, where the second messenger cyclic 2’, 3’-GMP-AMP (hereafter referred to as cGAMP) orchestrates the induction of type I interferons (IFNs) to combat viral infections. Despite its therapeutic potential, the clinical translation of cGAMP is severely hampered by its intrinsic physicochemical properties—high hydrophilicity and polyanionic charge—which result in poor membrane permeability and suboptimal bioavailability. Here, we identify Pt5-1c, a zebrafish phosvitin-derived antimicrobial peptide, as a potent immunomodulatory agent that overcomes this delivery bottleneck. Our results demonstrate that Pt5-1c significantly amplifies cGAMP-mediated immune signaling by enhancing its extracellular availability. Mechanistically, Microscale Thermophoresis (MST) and LC-MS/MS analyses reveal that Pt5-1c directly complexes with cGAMP, facilitating its efficient delivery to target cells, thereby triggering a robust STING-dependent cascade and downstream effector expression. In both *in vitro* and *in vivo* models, the Pt5-1c/cGAMP synergy elicited a superior interferon response and conferred profound protection against virus challenges. This study not only uncovers a novel functional role for antimicrobial peptides as facilitators of intracellular signaling but also provides a strategic framework for the development of next-generation STING-targeted antiviral immunotherapies.

## 1 Introduction

Our previous research showed that zebrafish embryos have extremely high resistance to microbial infections (such as pathogenic *Aeromonas hydrophila*), which is mainly attributed to the high content of phosvitin (Pv) in zebrafish eggs (1). We found that the peptide (Pt5) composed of 55 amino acid residues at the C-terminus of this zebrafish Pv has antibacterial and immunomodulatory activity (2). Through truncation and amino acid substitution of the parent peptide, we developed a 29-amino acid derivative, Pt5-1c (3). Compared with the parent peptide, Pt5-1c exhibits stronger antibacterial activity and excellent inhibitory activity, including against multidrug-resistant (MDR) strain (3). Furthermore, susceptibility to antibiotics was restored by Pt5-1c against *S. aureus* USA500 (oxacillin and vancomycin), *E. coli* 577 (streptomycin), and *K. pneumoniae* 2182 (azithromycin) (4). Crucially, no antimicrobial resistance was induced after prolonged exposure to Pt5-1c (4). The above studies clearly demonstrated that Pt5-1c is a highly promising antimicrobial agent; however, its activity in terms of modulating host antiviral properties has not yet been studied.

The endogenous second messenger 2’, 3’-cyclic GMP-AMP (hereafter referred to as cGAMP) plays a key role in regulating the host’s antiviral immune response (5, 6). In mammalian cells, the activation of the cytosolic DNA signaling pathway leads to the synthesis of cGAMP from ATP and GTP, a reaction catalyzed by the enzyme cGAS (7). The synthesized cGAMP acts as a ligand for the stimulator of interferon genes (STING), promoting STING oligomerization and its subsequent trafficking through intracellular routes to the Golgi network (5, 7–10). This process ultimately activates the TBK1 kinase and IRF3 transcription factor, thereby initiating the efficient synthesis of type I interferons (IFNs) (5, 7–10). The critical role of cGAS-cGAMP-STING signaling in viral surveillance has motivated recent research to leverage cGAMP as a therapeutic to stimulate antiviral immunity (11–14). Although cGAMP, an anionic, highly water-soluble molecule, demonstrates promising therapeutic potential, its low bioavailability and poor transmembrane delivery efficiency limit the molecule’s biological activity and therapeutic efficacy (15, 16). Therefore, cGAMP struggles to effectively cross the plasma membrane and reach the cytoplasmic solute region where the STING protein resides. This significantly limits the immunomodulatory activity and antiviral activity of cGAMP.

In this study, we found that Pt5-1c can significantly amplify the immune signal of cGAMP. Pt5-1c efficiently promotes cGAMP activation of interferon responses and other downstream pathways in a STING-dependent manner. We employed intercellular transfer assays, liquid chromatography-tandem mass spectrometry (LC-MS/MS) analysis and Microscale Thermophoresis (MST) approach to investigate the mechanism by which Pt5-1c enhances cGAMP immune signaling. We demonstrated that Pt5-1c facilitates the delivery of cGAMP across the target cell membrane, leading to STING activation and subsequent amplification of immune signal. Pt5-1c facilitates this cGAMP transfer by binding to it and enhances the cGAMP-mediated robust IFN and other host defense responses against viruses both *in vitro* and *in vivo*. In summary, our work reveals how Pt5-1c functions, expands our understanding of the link between antimicrobial peptides and innate immunity, and suggests new therapeutic opportunities for viral infections.

## 2 Materials and methods

### 2.1 Cell culture

RAW264.7 and THP‑1 cells were cultured at 37°C in a humidified 5% CO_2_ atmosphere. RAW264.7 cells were grown in DMEM (Gibco), while THP‑1 cells were maintained in RPMI-1640 (Gibco). Both media were supplemented with 10% FBS (Gemini) and 1% penicillin-streptomycin (Invitrogen), providing final concentrations of 100 U/mL penicillin and 100 μg/mL streptomycin.

### 2.2 Animal model

Female C57BL/6 mice (6–8 weeks old, weighing 20 ± 2.0 g) were procured from Charles River Laboratories (Beijing, China) and housed under specific pathogen-free (SPF) conditions with ad libitum access to food and water. All experimental procedures were approved by the Institutional Animal Care and Use Committee (IACUC) of China Agricultural University and were conducted in strict accordance with the Guidelines for the Care and Use of Laboratory Animals issued by the Ministry of Science and Technology of the People’s Republic of China.

### 2.3 Pt5-1c synthesis

The Pt5-1c compound, with a purity of approximately 95%, was synthesized and purified by GL Biochem Ltd. (Shanghai, China). The peptide was dissolved in endotoxin-free water, aliquoted, and stored at -20°C for subsequent use.

### 2.4 STING^-/-^ cell line generation

The LentiCRISPR v2 plasmid (Addgene) was utilized to generate the knockout cell lines. According to the Lentiviral CRISPR Toolbox manual, the guide sequence targeting STING (ACTCTTCTGCCGGACACTTG) was cloned into a lentiviral vector. HEK293T cells were transfected with polyethylenimine (PEI) to package lentivirus using a combination of 14 μg lentiviral construct, 6 μg psPAX2, and 4 μg pMD2.G. Viral supernatants were harvested from producer cells after 72 hours and filtered through 0.45_μm filters. The filtered supernatant was then added to THP-1 cells.

### 2.5 Luciferase reporter assay

THP-1-Lucia ISG and RAW-Lucia ISG reporter cells were created by stably integrating a luciferase gene driven by five interferon-stimulated response elements (ISRE) into THP-1 and RAW264.7 cells, respectively. These cell models serve as biosensors for STING pathway activation, which is detected by measuring ISRE-induced luciferase activity. THP-1-Lucia ISG and RAW-Lucia ISG cells were treated with cGAMP (1 μM) and/or Pt5-1c (60 μg/mL) for 12 to 24 hours. ISRE-driven luciferase activity was subsequently measured in both cell lines.

### 2.6 Western blot analysis of protein expression and phosphorylation

THP-1 and RAW264.7 cells were treated with cGAMP (2 μM) and/or Pt5-1c (60 μg/mL) for one hour prior to lysis. The resulting lysates were resolved by SDS-PAGE and transferred onto PVDF membranes. After blocking with 5% non-fat milk for 2 hours, the membranes were incubated overnight at 4 °C with specific primary antibodies, followed by a 1-hour incubation with appropriate secondary antibodies. Protein signals were visualized via chemiluminescence and captured using a ChemiDoc MP system (Bio-Rad).

### 2.7 ELISA assay

To assess cytokine secretion, THP-1 cells were stimulated with cGAMP (1 μM) and/or Pt5-1c (60 μg/mL) for 12 hours. IFN-β and CXCL10 levels in the corresponding cell culture supernatants and serum samples were then analyzed using ELISA kits (Invitrogen).

### 2.8 Quantitative PCR

After treatment of THP-1 cells with cGAMP (1 μM) and/or Pt5-1c (60 μg/mL) for 4 hours, total RNA was extracted with TRIzol Reagent (Thermo Fisher Scientific) for subsequent cDNA synthesis using the iScript kit (Bio-Rad). Gene expression was quantified by real-time PCR with iTaq Universal SYBR Green Supermix (Bio-Rad), normalized to GAPDH, and calculated via the 2^_ΔΔCT method.

### 2.9 LC-MS/MS or functional assay for cGAMP quantification

HEK293T cells (deficient in endogenous cGAS) were transfected with a human cGAS plasmid or an empty vector. Following this, cells were incubated in fresh medium with or without 60 μg/mL Pt5-1c for 20 minutes. The concentration of cGAMP in the supernatant was subsequently measured by LC-MS/MS. Quantification was carried out by monitoring the transition pairs m/z 675.1-524.1 and m/z 675.1-506.1, which were selectively optimized for analyte detection. A cGAMP standard solution at 10 ng/mL served as the external calibrant.

Subsequently, the conditioned medium from donor cultures was transferred to acceptor cells capable of responding to cGAMP, cGAS^-/-^ THP-1-Lucia ISG cells for 24h, to monitor STING activation.

### 2.10 Fluorescence microscopy

To assess the cell-penetrating ability of Pt5-1c, cells were seeded in plates and cultured overnight. The cells were then incubated with FITC-labeled Pt5-1c at a concentration of 60 μg/mL for 10 min at 37°C. Following incubation, the cells were washed extensively with PBS three times to remove any non-internalized peptides. Cells were then fixed with 4% paraformaldehyde and nuclei were counterstained with DAPI. The intracellular fluorescence signals were observed and captured using a fluorescence microscope.

### 2.11 Microscale thermophoresis

The interaction strength between cGAMP and Pt5-1c was assessed via microscale thermophoresis (MST). Briefly, a series of cGAMP dilutions were prepared in phosphate-buffered saline (PBS), followed by the addition of 250 nM FITC-labeled Pt5-1c to each tube. A Monolith NT.115 Standard Treated Measurements were commenced by capillary immersion in respective samples. The interaction data were processed and fitted using the MO.Affinity Analysis software (NanoTemper) to determine binding parameters.

### 2.12 Viral infection and titer quantification

WT and STING^^-^/^-^^ THP-1 cells were infected with HSV-1-GFP or PRV-GFP (MOI = 1) in the presence or absence of cGAMP (4 μM) and/or Pt5-1c (60 μg/mL) for 24 hours. Viral infection efficiency was subsequently quantified via fluorescence microscopy using a Zeiss Stemi508 system.

The mice were randomly allocated into four groups: control, cGAMP, Pt5-1c, and Pt5-1c + cGAMP. One hour prior to intravenous HSV-1 challenge, we administered a pretreatment of 30 μg cGAMP and/or 60 μg Pt5-1c to the animals. Body weight changes were recorded daily throughout the experiment. After dissection, spleens from mice were harvested and mechanically disrupted using sterile homogenizers in ice-cold PBS. HSV titer was determined by plaque assay 24 h post-infection. Extraction of DNA from infected tissues was performed. Quantitative PCR (qPCR) was then employed to measure the expression of the HSV-1-specific genes polymerase (pol), glycoprotein D (gD), and glycoprotein B (gB).

### 2.13 Quantification and statistical evaluation

All data are presented as mean ± SD. Statistical analyses were conducted using one-way ANOVA in GraphPad Prism 8, with significance denoted as *p ≤ 0.05, **p ≤ 0.01, ***p ≤ 0.001, ****p ≤ 0.0001; differences with p > 0.05 were considered not significant (NS).

## 3 Results

To systematically investigate the immunomodulatory potential of the antimicrobial peptide Pt5-1c, we designed a step-wise experimental strategy. First, we evaluated whether Pt5-1c enhances cGAMP-mediated STING activation and downstream interferon responses. Next, we examined the strict dependence of these effects on the STING signaling pathway. To understand the underlying mechanism, we then investigated the direct binding between Pt5-1c and cGAMP, as well as the facilitation of cGAMP cellular uptake. Finally, we assessed the physiological relevance of these findings by evaluating the antiviral efficacy of the Pt5-1c/cGAMP combination both *in vitro* and *in vivo*.

### 3.1 Pt5-1c potentiates cGAMP-triggered STING signaling and elicits robust interferon responses

**Figure 1:**
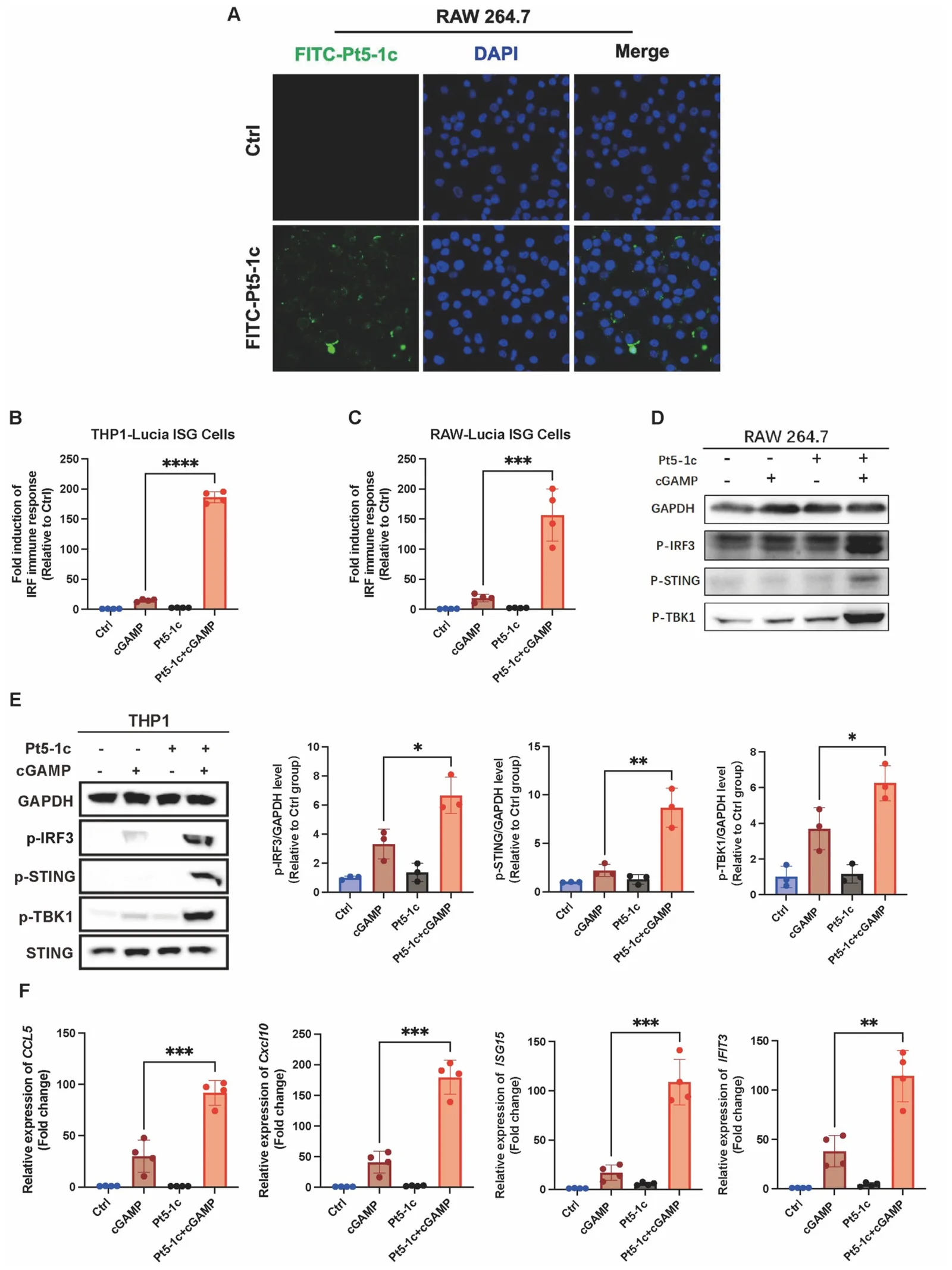
Pt5-1c augments cGAMP-induced STING signaling activation, leading to a strong interferon response. **(A)** Fluorescence microscopy images demonstrating the cell-penetrating ability of Pt5-1c. Cells were incubated with FITC-labeled Pt5-1c for 10 min, washed extensively, and imaged. Green indicates FITC-Pt5-1c, and blue indicates DAPI-stained nuclei. **(B)** THP‑1-Lucia ISG cells were incubated for 24 hours with cGAMP (1 μM) and/or Pt5-1c (60 μg/mL). **(C)** RAW-Lucia ISG cells were processed and evaluated using the method outlined in **(B)**. **(D)** Western blot analysis of STING signaling components in RAW264.7 cells. Cells were treated with cGAMP (2 μM) and/or Pt5-1c (60 μg/mL) for one hour. **(E)** Following a 1-hour treatment of THP‑1 cells with cGAMP (2 μM) and co-treatment with or without Pt5-1c (60 μg/mL), the protein levels of p-STING, p-TBK1, p-IRF3 and GAPDH were analyzed by measuring their band intensity with ImageJ. **(F)** Treatment of THP‑1 cells with cGAMP (2 μM) and/or Pt5-1c (60 μg/mL) was carried out for 6 hours. Measurement of CCL5, CXCL10, ISG15, and IFIT3 expression was subsequently performed via qPCR. In all panels, the control group (Ctrl) refers to cells treated with an equal volume of PBS without cGAMP or Pt5-1c. Statistical significance was determined by one-way ANOVA (n = 4 biological replicates). Data are representative of 4 independent experiments. (*p ≤ 0.05, **p ≤ 0.01, ***p ≤ 0.001, and ****p ≤ 0.0001).

To exert its intracellular effects, Pt5-1c must first cross the host cell membrane. To investigate its cell-penetrating ability, we incubated mammalian cells with FITC-labeled Pt5-1c. Fluorescence microscopy revealed strong intracellular FITC signals that persisted even after extensive washing, directly confirming the intrinsic capacity of Pt5-1c to freely traverse the cellular membrane (**Figure 1A**). When treated with cGAMP alone, the increase in the IFN-stimulated response element (ISRE) reporter activity was relatively limited in both THP‑1-Lucia ISG cells and RAW-Lucia ISG cells (**Figures 1B, C**). Pt5-1c treatment significantly boosted cGAMP-induced ISRE activity in THP‑1-Lucia ISG and RAW-Lucia ISG cells compared to cGAMP alone (**Figures 1B, C**). Exposure of wild type (WT) THP‑1 cells to cGAMP and Pt5-1c together significantly increased the phosphorylation of TBK1, STING, and IRF3 (**Figure 1E**). To confirm these findings in a murine model, we performed similar experiments in RAW264.7 cells. Consistent with the results in THP-1 cells, the co-treatment significantly augmented the activation of STING signaling components in RAW264.7 cells (**Figure 1D**). The results indicated that synergistic effects depend on the TBK1-STING-IRF3 phosphorylation cascade upstream of nuclear transcription signals. The expression of cGAMP-induced immune cytokines (ISG15, IFIT3) and chemokines (CXCL10, CCL5) in the STING pathway was significantly and consistently enhanced by Pt5-1c (**Figure 1F**). In summary, these findings identify Pt5-1c as a potent potentiator of cGAMP-triggered STING activation and interferon production.

### 3.2 Pt5-1c aids cGAMP to enhance interferon responses in a STING-dependent manner

**Figure 2:**
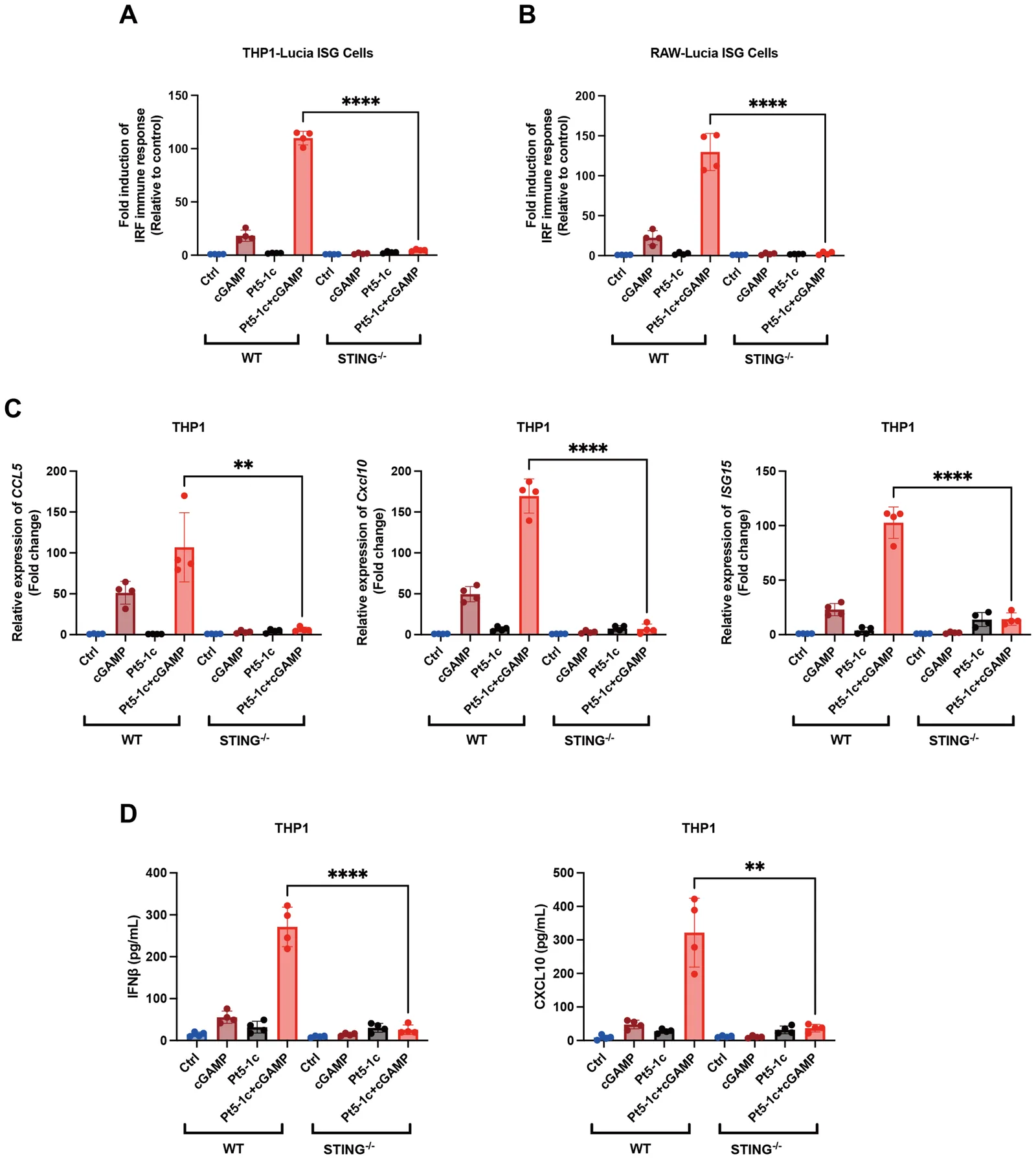
STING is required for Pt5-1c–cGAMP to activate TBK1-IRF3 signaling and the subsequent interferon response. **(A)** WT and STING^-/-^ THP-1-Lucia ISG cells were incubated for 24 hours with cGAMP (1 μM) and/or Pt5-1c (60 μg/mL). **(B)** WT and STING^-/-^ RAW-Lucia ISG cells were processed and evaluated using the method outlined in **(A)**. **(C)** Treatment of WT and STING^-/-^ THP-1 cells with cGAMP (1 μM) and/or Pt5-1c (60 μg/mL) was carried out for 6 hours. Measurement of CCL5, CXCL10 and ISG15 expression was subsequently performed via qPCR. Untreated cells served as the reference control for normalization. **(D)** WT and STING^-/-^ THP-1 cells were incubated with cGAMP (1 μM) and/or Pt5-1c (60 μg/mL) for 12 hours. Levels of IFN-β and CXCL10 in the culture supernatants were quantified using ELISA. For all experiments, the control group (Ctrl) indicates WT or STING^-/-^ cells treated with an equal volume of PBS without cGAMP and Pt5-1c. Data are presented as mean ± SD. Statistical significance was determined by one-way ANOVA (n = 4 biological replicates). Data are representative of 4 independent experiments. (**p ≤ 0.01, ****p ≤ 0.0001).

STING CRISPR knockout (STING^-/-^) THP‑1-Lucia ISG cells were then generated. Experiments revealed that Pt5-1c significantly enhanced the cGAMP-mediated upregulation of ISRE reporter activity in WT THP‑1-Lucia ISG and RAW-Lucia ISG cells. However, this enhancement was completely abolished in STING^-/-^ cells. This confirms that Pt5-1c mediates cGAMP activation of the TBK1-IRF3 signaling pathway to induce an interferon response (**Figures 2A, B**). Pt5-1c significantly enhanced the expression of CXCL10, CCL5 and ISG15 in WT THP‑1 cells. However, genetic ablation of STING abrogated the influence of Pt5-1c on cGAMP-triggered gene expression of CXCL10, CCL5, and ISG15 relative to the untreated control (**Figure 2C**). Co-treatment with Pt5-1c and cGAMP elevated the production of IFN-β and CXCL10 in WT THP‑1 cells relative to cGAMP alone. In contrast, STING-deficient THP‑1 cells failed to respond to combined treatment with Pt5-1c and cGAMP (**Figure 2D**). Taken together, these findings demonstrate that a functional STING protein is required for Pt5-1c to trigger TBK1–IRF3 signaling through cGAMP and subsequently promote interferon production.

### 3.3 Pt5-1c forms a complex with cGAMP and propagates immune responses

**Figure 3:**
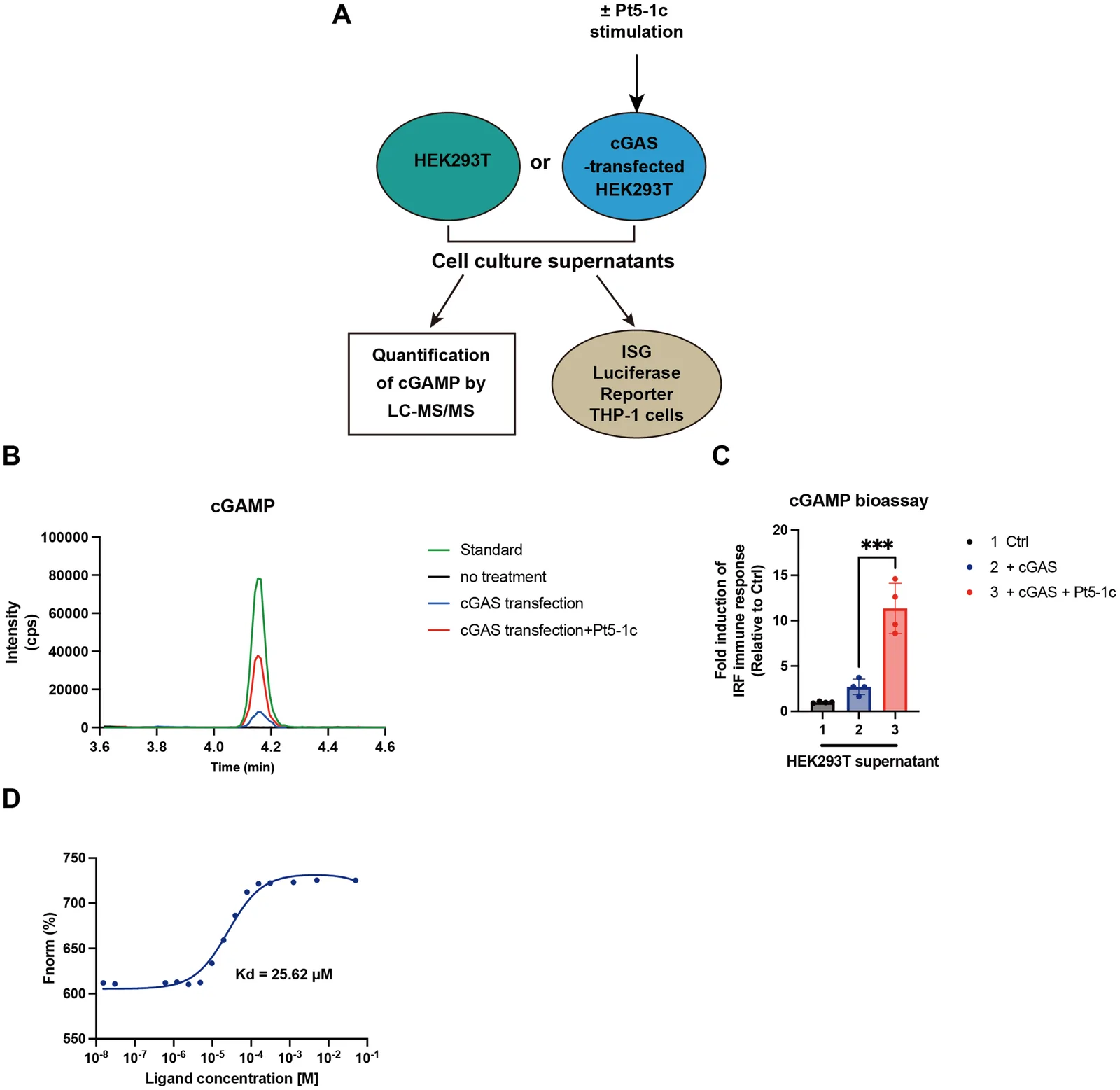
Pt5-1c facilitates the transfer of cGAMP and promotes immune activation through its direct interaction with cGAMP. **(A)** A schematic representation of the multicellular immune response assay induced by cGAMP. HEK293T cells were transfected with either a human cGAS expression vector or an empty vector control. Following transfection, the cells were incubated in fresh medium with or without Pt5-1c (60 μg/mL) for 20 minutes. Subsequently, cGAMP in the supernatant was measured by LC-MS/MS. These conditioned supernatants were then applied to THP-1-cGAS^-/-^ Lucia ISG cells to evaluate downstream STING activity. **(B)** cGAMP levels in the HEK293T supernatant were determined, with a 10 ng/mL cGAMP standard as a reference. **(C)** cGAS^-/-^ THP-1-Lucia ISG cells were exposed to supernatants harvested from HEK293T cell cultures for 24 h. The numbers on the x-axis correspond to the different supernatant treatments indicated in the panel legend (1 for Ctrl, 2 for +cGAS, and 3 for +cGAS + Pt5-1c). The control group (Ctrl, Group 1) represents cells treated with supernatants from HEK293T cells transfected with an empty vector and without Pt5-1c stimulation. Data are presented as mean ± SD. Statistical significance was determined by one-way ANOVA (n = 3 biological replicates). Data are representative of 3 independent experiments (***p ≤ 0.001). **(D)** The binding affinity between Pt5-1c and cGAMP was measured using MST. The MST experiment was independently repeated three times (n = 3).

As demonstrated by our fluorescence imaging data (**Figure 1A**), Pt5-1c is capable of traversing the cellular membrane freely. In order to assess whether Pt5-1c amplifies the immune response by facilitating the intercellular distribution of cGAMP from one cell to another, cGAS-deficient HEK293T cells were transfected with either a human cGAS expression plasmid or an empty control vector. Cells were then divided into two groups and exposed to Pt5-1c or left untreated for 20 minutes. Semi-automated LC-MS/MS analysis revealed a significantly higher concentration of cGAMP in the supernatant of Pt5-1c-treated HEK293T cells compared to untreated controls (**Figure 3B**). To assess STING activation, these cGAMP-enriched supernatants were applied to recipient cGAS^-/-^ THP‑1-Lucia ISG cells (**Figure 3C**). **Figure 3A** presents a schematic diagram illustrating the design of this multicellular immune response assay induced by cGAMP. The supernatant from Pt5-1c-treated HEK293T cells induced significantly stronger ISRE reporter activity than that from untreated cells (**Figure 3C**). In summary, these results demonstrate that Pt5-1c potentiates cGAS-triggered multicellular innate immune responses via facilitating intercellular cGAMP transfer.

In addition, to determine whether the facilitated delivery of cGAMP by Pt5-1c entails direct binding, MST was used to evaluate the direct binding of Pt5-1c to cGAMP (**Figure 3D**). The measured dissociation constant (Kd) between Pt5-1c and cGAMP was 25.62 ± 2.37 μM (**Figure 3D**), providing direct evidence that Pt5-1c facilitates cGAMP delivery via direct binding. Collectively, these findings support a model whereby Pt5-1c directly binds to cGAMP, enabling its intercellular transfer and subsequent propagation of immune activation.

### 3.4 Pt5-1c facilitates the cGAMP-mediated host defense against viruses in a STING-dependent manner

**Figure 4:**
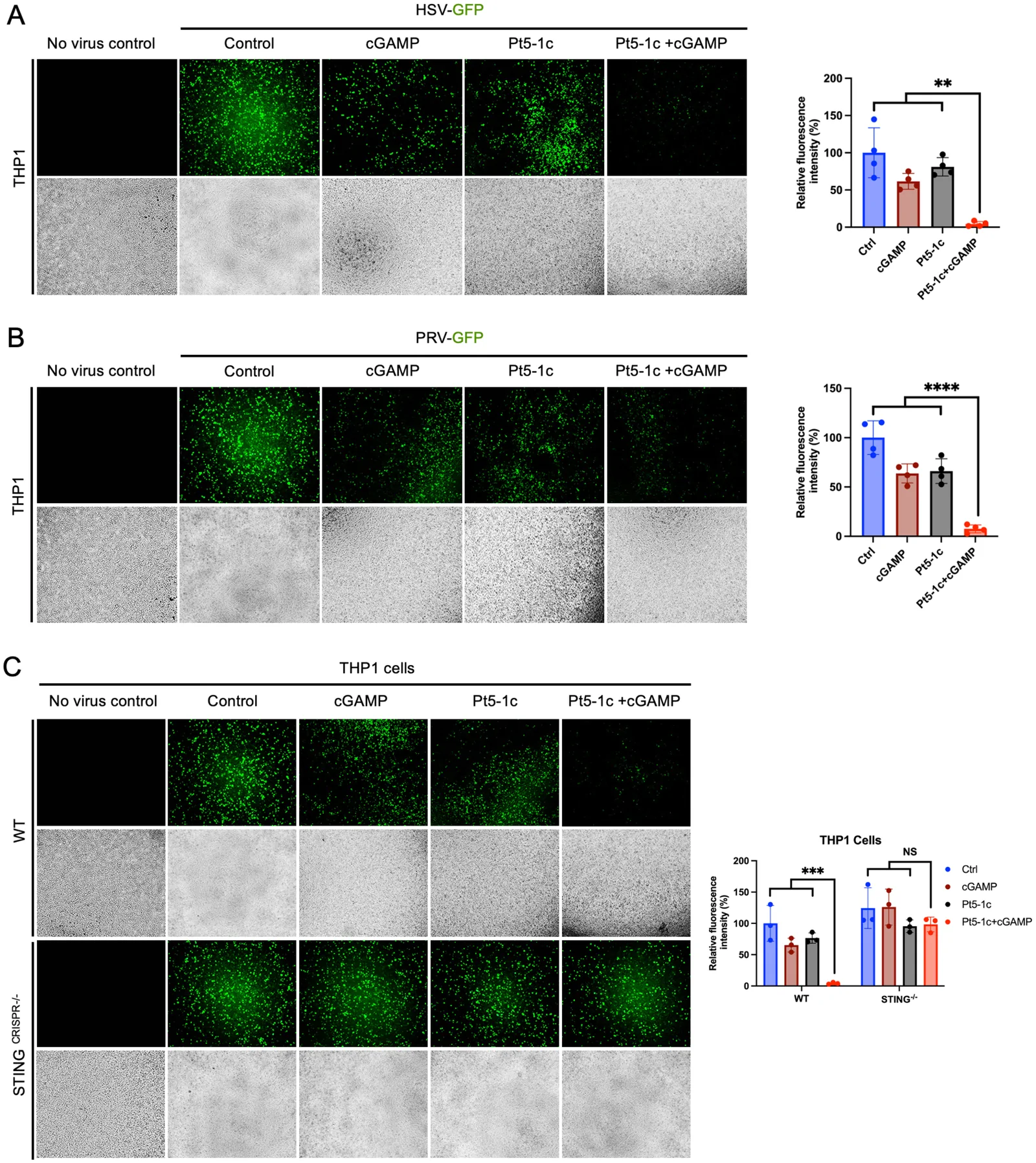
The potentiation of cGAMP-triggered antiviral immunity by Pt5-1c is dependent on the STING pathway. **(A)** THP-1 cells were pre-incubated for 4 hours with cGAMP and/or Pt5-1c before being challenged with HSV-1-GFP for 24 hours. Infection was assessed via fluorescence microscopy (scale bars = 100 μm), and HSV-1-GFP fluorescence intensity was quantified using ImageJ. Results are from three biological replicates (n = 3). **(B)** THP-1 cells were pre-incubated with cGAMP and/or Pt5-1c for 4 hours prior to infection with PRV-GFP for 24 hours. **(C)** WT and STING^-/-^ THP‑1-Lucia ISG cells were processed and evaluated using the method outlined in **(A)**. In **(A–C)**, the ‘No virus control’ refers to uninfected and untreated cells, while the ‘Control’ (Ctrl) group represents virus-infected cells treated with an equal volume of vehicle instead of cGAMP or Pt5-1c. Data are presented as mean ± SD. Statistical significance was determined by one-way ANOVA (n = 3 biological replicates). Data are representative of 3 independent experiments. (NS, p > 0.05; **p ≤ 0.01; ***p ≤ 0.001; ****p ≤ 0.0001).

To define the antiviral efficacy of Pt5-1c, HSV-1-GFP and PRV-GFP were used to infect THP‑1 cells. cGAMP or Pt5-1c alone only showed modest reduction of viral activity (**Figures 4A, B**). However, the limit of detection for viral replication was reached when cells were co-treated with Pt5-1c and cGAMP (**Figures 4A, B**). Following STING depletion, the antiviral activity of cGAMP was diminished, and the enhancing effect of Pt5-1c was lost (**Figure 4C**). The data support a model in which Pt5-1c potentiates cGAMP-mediated antiviral immunity via a STING-dependent mechanism.

### 3.5 Pt5-1c enhances cGAMP-associated antiviral responses *in vivo*

**Figure 5:**
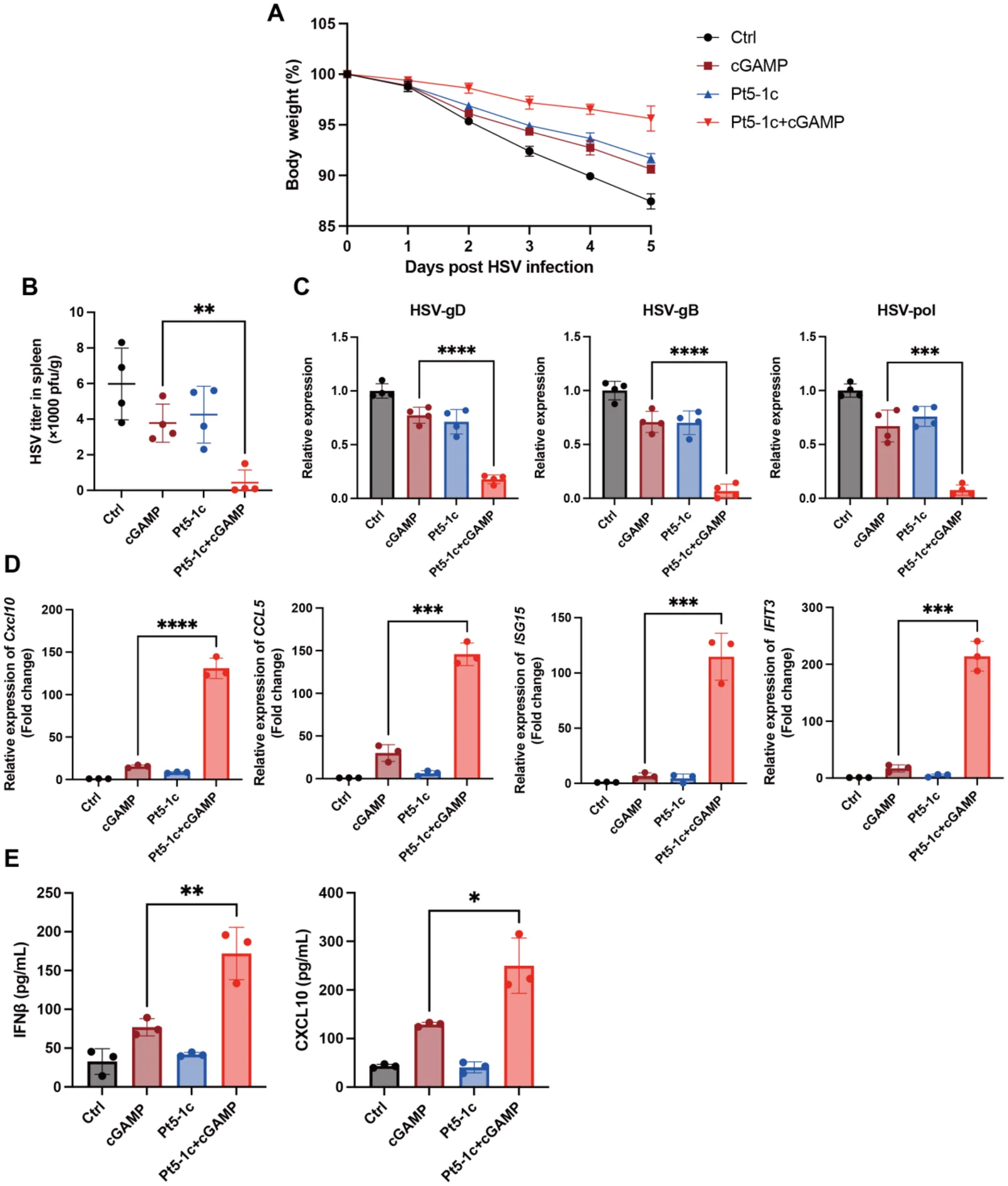
Pt5-1c enhances cGAMP-associated antiviral responses *in vivo*. **(A)** Prior to intravenous administration of HSV-1 (1 × 10^7^ PFU per mouse), mice were administered cGAMP and/or Pt5-1c for 1 hour. Daily body weight measurements were conducted, and there were three mice per group (n=3). **(B)** According to the procedure outlined in panel A, spleens were excised from the mice and processed into homogenates. The viral load of HSV in these samples was quantified using a plaque assay, with three biological replicates included per group. **(C)** Following the treatment protocol detailed in panel A, viral gene expression levels in spleen tissues were measured by quantitative PCR (qPCR), with a group size of n=3. **(D)** Mice were administered cGAMP and/or Pt5-1c over a 6-hour period. Subsequently, RNA was purified from tissues for the assessment of gene expression levels, with three biological replicates included per group. **(E)** In accordance with panel **(D)**, mice were treated and serum cytokine concentrations (IFN-β and CXCL10) were determined (n=3/group). In all animal experiments **(A–E)**, the control group (Ctrl) consists of HSV-1-infected mice administered with an equal volume of PBS instead of cGAMP or Pt5-1c. Statistical significance was determined by one-way ANOVA (n = 3 biological replicates). Data are representative of 3 independent experiments (*p ≤ 0.05, **p ≤ 0.01, ***p ≤ 0.001, and ****p ≤ 0.0001).

The limit of detection for viral replication was attained following treatment with both Pt5-1c and cGAMP. As depicted in **Figure 5A**, the combined administration of Pt5-1c and cGAMP demonstrated enhanced efficacy in protecting the host from HSV-induced weight loss compared to either agent alone. Furthermore, the combined treatment with Pt5-1c and cGAMP nearly eradicated viral infection in the spleens of mice (**Figures 5B, C**). Pt5-1c also potently enhanced the cGAMP-induced expression of ISG genes such as CXCL10, CCL5, ISG15, and IFIT3 (**Figure 5D**). ELISA results also consistently showed that the combination of Pt5-1c and cGAMP synergistically elevated serum levels of IFN-β and CXCL10 (**Figure 5E**). In conclusion, these data suggest that Pt5-1c augments cGAMP- associated IFN responses against viruses in mice. These findings collectively indicate that the Pt5-1c–cGAMP complex exhibits promising potential for application in immunotherapy against infectious diseases.

## 4 Discussion

Antimicrobial peptides (AMPs) are widely found in nature, including in plants, animals, and microbes (17). These peptides are typically essential components of the innate immune system, acting as crucial component in defending against various pathogenic microorganisms (18–20). However, despite extensive research on antimicrobial peptides, studies investigating their interactions with innate immune pathways — particularly novel signaling pathways such as cGAS-STING — remain limited. Here, we demonstrate that Pt5-1c, a zebrafish phosvitin-derived antimicrobial peptide, functions as an efficient facilitator. It facilitates the transmembrane delivery of cGAMP into target cells, thereby activating the cGAS-STING immune signaling pathway. Our findings reveal a novel mechanism and thereby establish a strategic approach for modulating cGAMP–STING-mediated immune responses.

cGAMP functions as a crucial intracellular second messenger in innate immune signaling (5, 6). The cGAS enzyme synthesizes cGAMP upon detecting signals of microbial invasion or cellular damage. Subsequently, cGAMP binds to STING, inducing activation of the STING pathway, which leads to the synthesis of type I interferons and proinflammatory cytokines—a process critical for antiviral and antitumor immunity (7–10, 21–23). However, cGAMP is negatively charged, and if present extracellularly, must depend on assistance to traverse the plasma membrane. This results in the bioavailability and immunomodulatory effects of cGAMP alone being extremely limited. In this study, we demonstrated that Pt5-1c markedly enhances cGAMP-mediated immune signaling. By binding to cGAMP, Pt5-1c promotes the delivery of cGAMP across the cell membrane, thereby enhancing STING-dependent interferon production and subsequent antiviral immunity. Crucially, the robust immunomodulatory effects observed *in vitro*, such as the upregulated expression of key chemokines and ISGs, were fully recapitulated in our *in vivo* HSV-1 infection model. This *in vivo* enhancement of systemic interferon and local antiviral gene expression confirms that the therapeutic synergy between Pt5-1c and cGAMP is maintained under actual viral infection conditions. These findings reveal a crucial function of Pt5-1c in innate immune activation and pave the way for novel antiviral therapeutic strategies. Although our data demonstrate the binding of Pt5-1c to cGAMP, this interaction is likely driven in part by electrostatic forces. Consequently, Pt5-1c may also interact non-specifically with other anionic nucleotides and nucleic acids, such as dsDNA. Furthermore, although we observed an increase in extracellular cGAMP, mild membrane perturbation or cytosolic leakage in cell models cannot be entirely excluded and may contribute to this phenomenon.

Many animals (such as fish, chickens, and mosquitoes) release their eggs externally for fertilization. Consequently, the resulting embryos and larvae are exposed to natural environments teeming with potential pathogens that can cause various diseases. This implies that in addition to nutrients, the egg should also contain immune defenses to protect the embryo from being infected by pathogenic microorganisms. Research has indicated that chicken Pv, a major component of yolk proteins, achieves ion chelation through its abundant phosphorylated serine residues, thereby effectively inhibiting the growth of Escherichia coli (24). Furthermore, recent findings demonstrate that mosquito vitellogenin precursor interferes with the immune response of Anopheles gambiae to Plasmodium infection (25). Similarly, we and our collaborators previously discovered that early zebrafish embryos exhibit antimicrobial activity against microorganisms, including pathogenic aquatic *Aeromonas hydrophila*, and that this activity correlates with the nutritional protein phosvitin (Pv) in the eggs (1). We found that the Pt5-1c peptide, which is derived from phosvitin, exhibits broad-spectrum antibacterial properties and effectively inhibits biofilm formation. It demonstrated synergistic effects with traditional antibiotics and exhibits significant antibacterial activity against multiple bacterial strains, including multidrug-resistant (MDR) strains (1–4). Here, we demonstrated that Pt5-1c significantly enhances cGAMP-dependent antiviral host defense. Pt5-1c likely functions as a key intercellular facilitator of cGAMP and is therefore critical for initiating innate antiviral immune responses. This may explain why fish embryos or larvae can thrive in aquatic environments teeming with bacteria and viruses.

In the clinical treatment of infected wounds, it is essential not only to inhibit the pathogen proliferation at the wound site but also to accelerate wound healing. Previous studies have demonstrated that Pt5-1c exhibits excellent antibacterial activity (1–4). In this study, we further demonstrated that Pt5-1c also boosts immune defenses and antiviral activity. Pt5-1c has also been shown to enhance fibroblast migration *in vitro*, as evidenced by scratch assay results, and to promote wound healing and epithelialization in murine dermal wound models *in vivo (*26). Pt5-1c enhances keratinocyte migration and proliferation through EGFR-dependent activation of the Akt/MAPK/STAT3 signaling pathways (26). This indicates that Pt5-1c peptide holds significant potential in treating wounds caused by burns or lacerations.

In summary, we show that the antimicrobial peptide Pt5-1c mediates intercellular cGAMP transfer and thereby stimulates STING-dependent activation of innate immunity. The data suggest that Pt5-1c is a compelling candidate as a novel immunomodulator, establishing a robust basis for its expanded application in clinical and pharmaceutical contexts.

## Data Availability

The raw data supporting the conclusions of this article will be made available by the authors, without undue reservation.
